# Molecular Mechanisms, Diagnosis, and Therapeutic Strategies of Antifungal Resistance in Filamentous Fungi

**DOI:** 10.3390/jof12080565

**Published:** 2026-08-01

**Authors:** Yajing Yin, Yueru Zhao, Meng Zhao, Lipeng Zhang, Ruijie Li, Lei Liu

**Affiliations:** 1 Tianjin Key Laboratory of Food Biotechnology, College of Biotechnology and Food Science, Tianjin University of Commerce, Tianjin 300134, China; 16632869596@163.com (Y.Z.); zhanglp@tjcu.edu.cn (L.Z.); a2757098275@163.com (R.L.); a15611185472@163.com (L.L.); 2 Universe Gene Technology (Tianjin) Co., Ltd., Tianjin 300134, China; zhaomeng19921207@163.com

**Keywords:** filamentous fungi, antifungal resistance, resistance mechanisms, molecular diagnostics, combination therapy

## Abstract

Antifungal resistance in filamentous fungi has emerged as a major threat to global public health, posing a serious challenge particularly to immunocompromised populations. This review provides a systematic overview of the molecular mechanisms, diagnostic approaches, and clinical therapeutic strategies for antifungal resistance in filamentous fungi, with a focus on *Aspergillus fumigatus*, *Fusarium* spp., *Mucorales*, and *Scedosporium* spp./*Lomentospora prolificans*. Resistance mechanisms can be broadly categorized as intrinsic resistance and acquired resistance. Intrinsic resistance arises from species-specific genetic traits, such as structural differences in target sites, constitutive overexpression of efflux pumps, and metabolic pathway redundancy. Acquired resistance develops under drug pressure through target gene mutations (e.g., hotspot mutations and promoter tandem repeats in *CYP51A*), efflux pump overexpression, biofilm formation, and epigenetic regulation. For diagnosis, conventional culture and antifungal susceptibility testing remain the gold standard; however, molecular techniques—including MALDI-TOF MS, targeted resistance gene PCR, and metagenomics—are substantially improving detection efficiency. Therapeutic strategies should be stratified based on antifungal susceptibility testing results and species identification. Precision dosing guided by therapeutic drug monitoring, combination therapy, and the introduction of novel agents (including isavuconazole, rezafungin, fosmanogepix, and olorofim) are progressively improving clinical outcomes. Looking ahead, global surveillance and multisectoral collaboration are essential to deepen our understanding of resistance evolution, accelerate the clinical translation of novel diagnostic and therapeutic tools, and curb the global spread of resistance.

## 1. Introduction

Filamentous fungi serve as primary decomposers of organic matter, function as “manufacturing factories” for food products and antibiotics, and represent eukaryotic model systems for scientific research. However, many of these species are also significant opportunistic pathogens in humans. With the expanding population of immunocompromised patients, including the increasing prevalence of HIV/AIDS, the growing use of hematopoietic stem cell transplantation and solid organ transplantation, cancer chemotherapy, and the widespread application of immunosuppressive agents, the incidence and mortality rates of invasive filamentous fungal infections have exhibited a marked upward trajectory. This trend has consequently emerged as a major challenge in global public health [1,2,3,4]. An estimated over 1.5 million life-threatening fungal infections occur worldwide each year, among which filamentous fungal infections account for a substantial proportion and are associated with persistently high mortality rates [5]. Among the numerous pathogenic filamentous fungi, invasive aspergillosis caused by the *A. fumigatus* complex is the most prevalent and constitutes a leading cause of fatal infections in patients with hematological malignancies and transplant recipients, with mortality rates frequently ranging from 30% to 80% [6]. Additionally, infections caused by non-*A. fumigatus* spp. (such as *A. niger*, *A. terreus*, *Fusarium* spp., *Mucorales* [e.g., *Rhizopus*, *Mucor*], and *Scedosporium* spp./*L. prolificans*) have been increasingly reported. These infections are notoriously difficult to diagnose, present with limited therapeutic options, and are associated with a dismal prognosis [2,7,8,9,10]. During the COVID-19 pandemic, the reported prevalence of concomitant pulmonary aspergillosis in Europe ranged from 20% to 35%, with an overall mortality rate of 63%. Moreover, the mortality rate of COVID-19-associated mucormycosis during an outbreak in Egypt reached 91%. These alarming figures have thrust the devastating impact of filamentous fungal infections into the public spotlight, underscoring the urgent need for effective prevention and therapeutic strategies [11,12].

In recent years, the emergence and spread of antifungal resistance—particularly the dissemination of triazole-resistant *A. fumigatus* isolates—have further compounded the clinical management of these infections. Acquired infections caused by environmental clonal isolates of azole-resistant *A. fumigatus* have been reported across multiple continents, including Europe, the Americas, and Oceania, with resistance rates exceeding 20% at certain medical centers. This trend has been associated with increased treatment failure and elevated mortality rates [13,14]. Of even greater concern, antifungal resistance has assumed a global dimension and is progressively spreading from *A. fumigatus* to other filamentous fungi. For instance, multidrug-resistant isolates of *Fusarium* spp., *Scedosporium* spp./*L. prolificans*, and *Mucorales* have all been documented [15,16,17]. Furthermore, resistance not only compromises the efficacy of existing therapeutic agents but also places prophylactic strategies at risk of failure, thereby giving rise to a complex and formidable multidrug resistance threat.

Given the urgency and complexity of antifungal resistance in filamentous fungi, a systematic synthesis of known resistance mechanisms and their clinical implications is urgently needed. Although numerous studies have addressed resistance to specific drugs or individual pathogens, an integrated review covering the latest molecular mechanisms, epidemiology, diagnostics, and therapeutic strategies is still lacking. This review provides a comprehensive overview of intrinsic and acquired resistance mechanisms across the major pathogenic genera—*A. fumigatus*, *Fusarium* spp., *Mucorales*, and *Scedosporium* spp./*L. prolificans*—and then surveys recent advances in diagnostic technologies. Based on mechanistic insights, we evaluate current therapeutic approaches and outline future research directions. Ultimately, this review aims to offer an up-to-date reference for clinical practice, antifungal drug development, and public health management, while promoting multidisciplinary collaboration to counter this escalating global threat.

## 2. Clinical Antifungal Agents: Mechanisms of Action and Resistance Challenges

**Figure 1:**
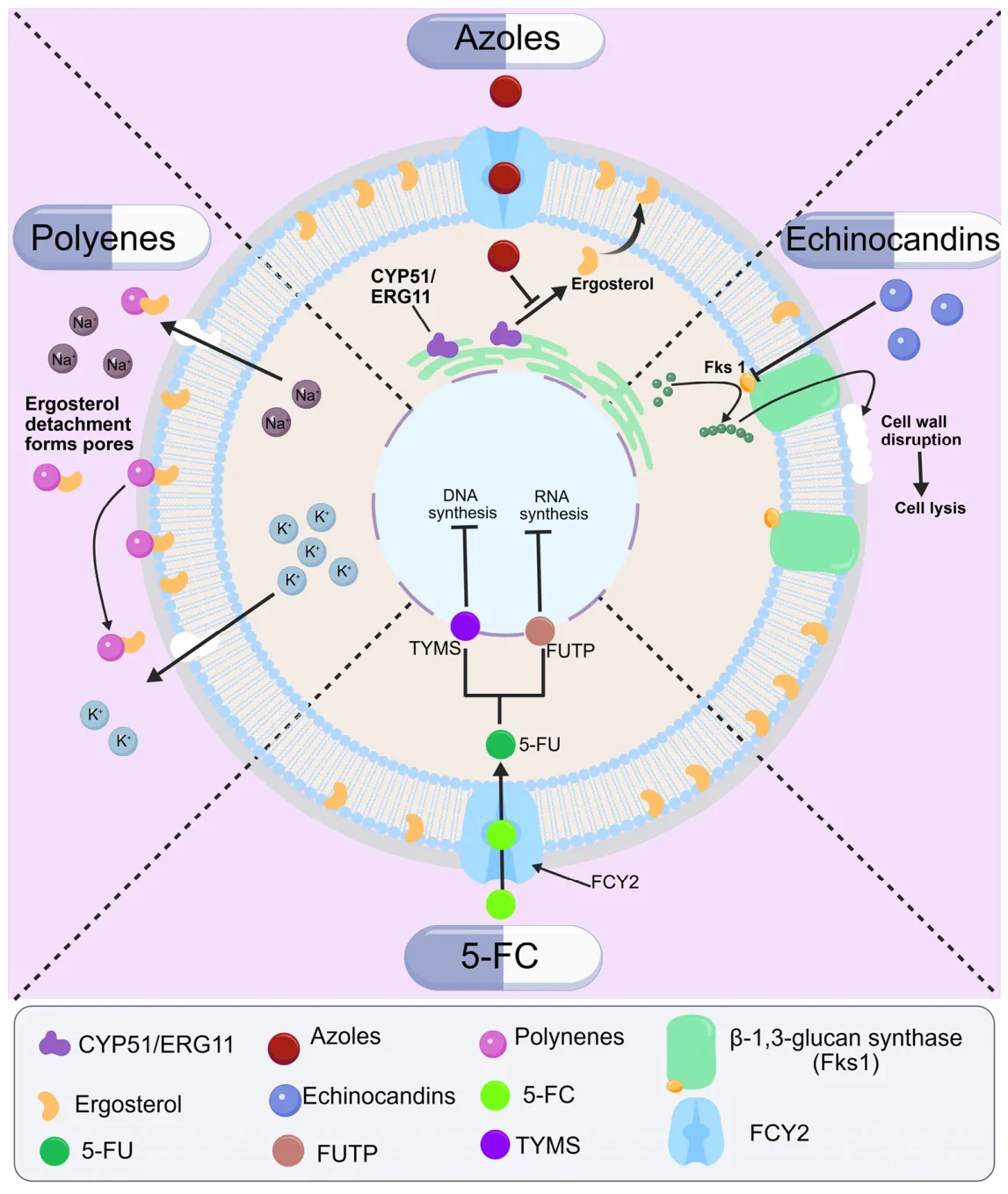
Mechanisms of action of major antifungal agents against filamentous fungi. Schematic representation of the cellular targets of the 4 main classes of antifungal drugs used in the treatment of invasive fungal infections. Azoles inhibit lanosterol 14α-demethylase (CYP51/ERG11), thereby blocking ergosterol biosynthesis in the cell membrane. Polyenes bind directly to ergosterol and form transmembrane pores, leading to cytoplasmic leakage and cell death. Echinocandins non-competitively inhibit β-(1,3)-D-glucan synthase, impairing cell wall integrity. Flucytosine (5-FC) is taken up via cytosine permease (FCY2) and deaminated to 5-fluorouracil (5-FU), which is further converted to 5-fluorodeoxyuridine monophosphate (5-FdUMP); this metabolite inhibits thymidylate synthase (TYMS), blocking DNA synthesis, while 5-FU is also converted to 5-fluorouridine triphosphate (5-FUTP), disrupting RNA synthesis. Key enzymes, metabolites, and drug targets are indicated.

Given the undeniable severity of fungal threats, a range of antifungal agents have been successively developed. Currently, the antifungal drugs available for clinical use against invasive fungal infections are primarily categorized into four classes—polyenes, azoles, echinocandins, and flucytosine—each of which possesses distinct molecular targets and is associated with corresponding resistance mechanisms (Figure 1) [18].

Azoles, represented by voriconazole, posaconazole, and isavuconazole, exert their fungicidal/fungistatic activity through inhibition of lanosterol 14α-demethylase—a key enzyme in the ergosterol biosynthesis pathway of the cell membrane—encoded by the *CYP51* gene. Voriconazole has long been the drug of choice for the treatment of invasive aspergillosis, while posaconazole and isavuconazole have also played important roles in both therapeutic and prophylactic settings [18,19,20]. However, it is precisely the widespread and prolonged use of azoles in both clinical settings and agriculture (as fungicides) that has contributed to the most prominent resistance issues. For example, azole-resistant *Aspergillus* isolates are widely distributed and have been documented on all continents except Antarctica [21]. *Fusarium* spp., *Mucorales*, and *Scedosporium* spp./*L. prolificans* are intrinsically resistant to azoles, and the azole resistance in clinical isolates has become increasingly severe in recent years [15,17,22].

The widespread use of azole fungicides in agriculture represents a major driver of environmental resistance selection. Triazole compounds such as tebuconazole, propiconazole, and epoxiconazole share structural and mechanistic similarities with clinical azoles, enabling cross-resistance in environmental *A. fumigatus* isolates. The emergence of TR34/L98H and TR46/Y121F/T289A genotypes has been unequivocally linked to agricultural fungicide exposure, with these mutations detected in soil, plant debris, and air samples from agricultural regions across Europe, Asia, and the Americas [23,24,25]. This environmental reservoir poses a unique challenge: even antifungal-native patients can acquire resistant infections through inhalation of resistant spores, as documented in multiple case series from regions with intensive fungicide use [21].

Polyenes, represented by amphotericin B (AmB) and its lipid formulations, exert their antifungal activity by binding to ergosterol in the fungal cell membrane, forming transmembrane channels that ultimately lead to cell death due to leakage of cellular contents. They exhibit broad-spectrum activity against a wide range of filamentous fungi and retain good activity against *Mucorales* [26]. However, severe adverse effects, including nephrotoxicity and infusion-related reactions, have restricted their long-term application at high doses [27]. Although resistance to AmB is not widespread, important exceptions exist, such as the intrinsic resistance observed in *A. terreus* [28]. Additionally, certain isolates of *Fusarium* spp. and *Scedosporium* spp./*L. prolificans* have also exhibited pan-resistance to this agent [29,30].

Echinocandins, such as caspofungin and micafungin, act through non-competitive inhibition of β-(1,3)-D-glucan synthase, thereby disrupting cell wall integrity [31]. They exhibit fungistatic activity against *Aspergillus* spp. and are frequently used as part of combination therapy; however, they are intrinsically inactive against most other filamentous fungi, including *Mucorales*, *Fusarium*, and *Scedosporium* spp./*L. prolificans* [15,16,17,32].

Flucytosine, a pyrimidine analogue, consists of a structural analogue of 5-fluorouracil. After deamination to 5-fluorouracil, it is subsequently converted to 5-fluorodeoxyuridine monophosphate, which inhibits thymidylate synthase and interferes with DNA synthesis. In addition, 5-fluorouracil is also converted to 5-fluorouridine triphosphate, which disrupts RNA synthesis. Flucytosine has a narrow spectrum of activity, and the emergence of resistance is highly frequent when used as monotherapy; therefore, it is only administered in combination with AmB for the treatment of certain deep-seated invasive fungal infections. However, its considerable host toxicity has rendered its clinical use extremely limited [18].

In addition to the four classes described above, several other antifungal compounds are available. For example, terbinafine, an allylamine and thiocarbamate compound, inhibits squalene epoxidase (Erg1p), thereby blocking ergosterol biosynthesis. It is commonly used in the treatment of dermatophytosis; however, monotherapy is prone to the rapid emergence of resistance, and it is therefore frequently employed as part of combination regimens [33].

Importantly, the molecular targets of these agents are relatively limited, and fungi are capable of evading their lethal effects through multiple evolutionary mechanisms. The emergence of resistance is driven not only by drug pressure selection during clinical therapy but also by the extensive use of fungicides in the environment—particularly in agriculture—that are structurally similar to clinical antifungals. This environmental selection pressure is now considered to be a major evolutionary force fueling the development and dissemination of resistance mutations [34,35]. This has made antifungal resistance a multifaceted issue that cuts across the disciplines of medicine, agriculture, and environmental science.

## 3. Major Pathogenic Filamentous Fungi and Their Resistance Profiles

### 3.1. Aspergillus spp.

#### 3.1.1. *Aspergillus* spp.

*A. fumigatus* is a major pathogen responsible for opportunistic fungal diseases in humans. According to the most recent estimates, it accounts for more than 50% of invasive fungal diseases worldwide and has been designated as a critical priority pathogen by the World Health Organization (WHO) in its Fungal Priority Pathogens List (FPPL) [36]. It primarily causes invasive pulmonary aspergillosis, chronic pulmonary aspergillosis, and allergic bronchopulmonary aspergillosis. Among these, invasive pulmonary aspergillosis is the most severe manifestation and predominantly affects immunocompromised individuals [37]. Aspergillosis is typically treated with triazole antifungals; however, owing to the intrinsic resistance of *A. fumigatus* to fluconazole, voriconazole has become the first-line therapeutic agent [38]. With the widespread use of azoles in both clinical and agricultural settings, *Aspergillus* spp. have developed resistance to this class of antifungals. In the late 1980s, the first strains of *Aspergillus* resistant to itraconazole were identified in the United States [39]. Subsequently, in 1997, azole-resistant *Aspergillus* isolates were also reported in Europe [40,41,42,43]. A year later, isolates carrying the G54 mutation and exhibiting resistance to azoles emerged in Japan [44]. Since then, studies on azole resistance have increased considerably worldwide and azole-resistant isolates have been documented on every continent with the exception of Antarctica [21].

#### 3.1.2. Non-*Fumigatus Aspergillus* spp.

Non-*fumigatus Aspergillus* spp., such as *A. flavus*, *A. terreus*, *A. niger*, and *A. nidulans*, have become more frequently detected in clinically relevant specimens. Among these, *A. flavus* is the second most common causative agent of invasive fungal diseases and is particularly prevalent in subtropical regions [45,46], It frequently causes invasive pulmonary aspergillosis and is also a common causative agent of invasive rhinosinusitis and external otitis in immunocompromised patients [47,48]. *A. nidulans* is the second most common causative agent of invasive fungal infections in patients with chronic granulomatous disease (CGD) [49]; in contrast, it is rarely recognized as a causative agent of invasive fungal disease in other immunocompromised patient populations [50]. *A. niger* is more commonly associated with milder clinical presentations, such as otomycosis [51]. Although the incidence of invasive aspergillosis caused by *A. niger* remains relatively low, it has nevertheless shown an upward trend [52]. Furthermore, the survival rate associated with *A. niger* infections is higher than that for *A. fumigatus* and *A. flavus* [53]. *A. terreus*, which exhibits intrinsic resistance to AmB, is emerging as an increasingly important fungal pathogen in invasive aspergillosis. A multinational surveillance study reported a prevalence of 5.2% for *A. terreus* among aspergillosis cases [54]. Resistance studies have shown that, in addition to intrinsic resistance to polyenes, clinical isolates of *A. flavus* and *A. terreus* with resistance to azoles have also been reported [45]. Elevated MICs to multiple azoles have also been observed in *A. niger*, and biofilm formation is hypothesized to be a contributing factor [55].

### 3.2. Fusarium spp.

Human fusariosis is a re-emerging disease worldwide, with its incidence having increased markedly since the 1970s and 1980s [56]. Infections caused by *Fusarium* spp. in humans are broadly categorized into superficial infections (e.g., keratitis, onychomycosis) and invasive infections, including fungemia and disseminated fusariosis in immunocompromised patients. In tropical regions, *Fusarium* is the leading causative agent of fungal keratitis, and the mortality rate associated with invasive fusariosis is considerably high [22]. In the clinical setting, *Fusarium* spp. show progressively increasing tolerance to nearly all first-line antifungal agents, positioning it as a “high-priority” human pathogen [16,57]. Despite such antifungal resistance, AmB and voriconazole remain the mainstay of treatment for invasive fusariosis; however, therapeutic decisions are contingent upon antifungal susceptibility testing of clinical isolates [58].

### 3.3. Mucorales

The order *Mucorales* comprises a group of filamentous fungi that includes 55 genera and 261 species, of which approximately 38 species have been documented to cause human infections. The most frequently encountered genera are *Rhizopus*, *Mucor*, *Lichtheimia*, and *Rhizomucor* [59]. *Mucorales* primarily cause cutaneous infections, rhino-orbital-cerebral mucormycosis, pulmonary mucormycosis, as well as gastrointestinal and disseminated mucormycosis. These infections exhibit a higher incidence in patients with diabetes mellitus, severely immunocompromised individuals, and those with a history of trauma, and are associated with notably high mortality rates [60,61]. Studies have shown that *Mucorales* possess intrinsic resistance to commonly used antifungal agents. For instance, they exhibit intrinsic resistance to short-tailed azoles, such as fluconazole and voriconazole. Echinocandins display weak in vitro activity against these fungi, with minimum inhibitory concentrations (MICs) up to 1000-fold higher than those observed for *Aspergillus* spp. and *Candida* spp. Flucytosine has been shown to exert no significant inhibitory effect in vitro, and terbinafine has demonstrated poor efficacy in animal models [62]. In vitro and in vivo susceptibility of *Mucorales* to medium-tailed azoles (e.g., isavuconazole) and long-tailed azoles (e.g., posaconazole) exhibits species-specific differences; therefore, these agents are only indicated for salvage therapy. In most cases, lipid formulations of AmB remain the treatment of choice [15].

### 3.4. Scedosporium spp./L. prolificans

*Scedosporium* spp. and *L. prolificans* (formerly known as *Scedosporium prolificans*) are non-*Aspergillus* filamentous fungi that have attracted increasing attention as causative agents of invasive fungal diseases (IFDs) in both immunocompromised and immunocompetent patients. They are categorized as “medium priority” pathogen in the first WHO FPPL [17]. In immunocompetent patients, *Scedosporium* spp./*L. prolificans* primarily cause localized infections, such as mycetoma of the foot, osteoarticular infections, or central nervous system (CNS) infections following near-drowning incidents. In immunocompromised patients, they predominantly cause pulmonary and CNS infections, fungemia, and disseminated disease [63]. Furthermore, owing to the limited availability of effective therapeutic options, invasive infections caused by these fungi are associated with markedly high mortality rates [64]. In terms of antifungal tolerance, *Scedosporium* spp. typically exhibit high MICs to AmB, itraconazole, and isavuconazole, whereas voriconazole and posaconazole show favorable in vitro activity, albeit with notable inter-species variability. In contrast, *L. prolificans* is pan-resistant to most currently available antifungal agents, including AmB, azoles, and echinocandins; however, it has shown promising in vitro activity against novel agents such as olorofim [4].

## 4. Mechanisms of Antifungal Resistance in Filamentous Fungi

Antifungal resistance in filamentous fungi is a multidimensional and multilayered biological phenomenon, fundamentally stemming from the interplay between intrinsic physiological traits of the pathogen and adaptive responses under antifungal drug selection pressure. Resistance mechanisms can be systematically categorized into two broad classes: intrinsic (inherent) resistance and acquired resistance. Intrinsic resistance represents an inherent genetic trait of a given fungal taxon that does not require drug induction and thus constitutes the “baseline” challenge to antifungal therapy. Acquired resistance, in contrast, is a direct product of genomic or epigenomic alterations in the fungus under drug selective pressure and is the leading cause of clinical treatment failure [65]. In this chapter, we systematically dissect the core molecular mechanisms underlying the two classes of resistance described above, with a primary focus on *A. fumigatus* and other clinically relevant *Aspergillus* spp., *Fusarium* spp., *Mucorales*, and *Scedosporium* spp./*L. prolificans*. By integrating the latest research advances, we aim to construct an integrative conceptual framework that spanning from static structural determinants to dynamic regulatory networks, and from single-gene mutations to global network-level responses.

### 4.1. Intrinsic (Inherent) Resistance: Natural Genetic Traits

Intrinsic resistance establishes the initial efficacy baseline of antifungal agents and therefore constitutes a prerequisite constraint that must be considered first in both drug development and the selection of clinical therapeutic regimens. Due to their distinct biological characteristics, different genera and species of filamentous fungi exhibit intrinsically distinct resistance profiles.

#### 4.1.1. Intrinsic Resistance of *Aspergillus* spp.

The intrinsic insusceptibility of *A. fumigatus* to fluconazole is the fundamental cause of its failure as a therapeutic agent against this pathogen. Studies have identified the T289 residue of the target enzyme CYP51A as the critical determinant conferring fluconazole resistance in *A. fumigatus*. When this residue undergoes mutation (i.e., T289A), a marked increase in the level of fluconazole resistance is observed. Furthermore, the spatial region encompassing this residue—including the BC-loop—has been characterized as a crowded and conformationally sensitive structure that contributes to this innate resistance by influencing the binding of substrates and drugs. In addition, when T289A occurs in combination with another common mutation, Y121F, a synergistic effect has been demonstrated, resulting in high-level acquired resistance to fluconazole [66]. This further illustrates that fluconazole resistance in *A. fumigatus* represents a continuum that progresses from low-level intrinsic tolerance to high-level resistance achieved through specific combinations of mutations.

*A. terreus* exhibits intrinsic resistance to AmB, posing a substantial clinical challenge. Available evidence indicates that this resistance is multifactorial, resulting from the convergence of multiple mechanisms. (1) A strong stress response mediated by heat shock proteins. In AmB-resistant strains of *A. terreus*, the mRNA and protein levels of Hsp70 family members (Ssa and Ssb) are markedly upregulated upon AmB induction, whereas susceptible strains exhibit only a weak response. Notably, treatment with Hsp70 inhibitors substantially enhances the susceptibility of resistant strains to the drug [67]. Additionally, the interaction between Hsp90 and calcineurin also modulates the cellular stress response to AmB, and inhibition of their activity has been shown to significantly reduce the MIC of AmB against strains [68]. (2) Oxidative stress defense mechanisms. In resistant strains of *A. terreus*, mitochondrial copy number and oxygen consumption rates are reduced. Furthermore, upon AmB treatment, the activity of mitochondrial complex I does not show a significant enhancement; instead, the mitochondrial alternative oxidase (AOX) exhibits markedly increased activity, accompanied by reduced endogenous ROS generation. In addition, the activities of catalase (CAT) and superoxide dismutase (SOD) are enhanced in resistant strains, leading to an increased capacity for scavenging endogenous ROS and consequently mitigating oxidative damage [69]. (3) Other potential mechanisms. Recent studies have revealed that in resistant strains of *A. terreus*, the expression of P-type ATPase genes is significantly higher than that in their sectoring susceptible derivatives, and that mutations are present in polyketide synthase genes [70]. These findings may represent novel resistance-associated factors; however, further validation is warranted.

#### 4.1.2. Intrinsic Resistance of *Fusarium* spp.

*Fusarium* spp. generally exhibit elevated MICs to azoles, indicative of low susceptibility. First, *Fusarium* spp. possess three paralogous copies of the azole target enzyme lanosterol 14α-demethylase (CYP51), namely *CYP51A*, *CYP51B*, and *CYP51C*. Through functional complementation, these three genes increase the metabolic complexity of the sterol biosynthesis pathway, thereby providing a basis for intrinsic resistance to azoles [71]. Furthermore, the CYP51 proteins of *Fusarium* harbor known key site substitutions, which are hypothesized to contribute to intrinsic resistance. For example, in clinically relevant *Fusarium* spp., the *CYP51A*, *CYP51B*, and *CYP51C* gene regions harbor 6 key amino acid substitutions that have been documented in *A. fumigatus* as being associated with azole resistance. These include L98H, T289A, G448S/A, G138A, M220I/L/V, and N266T/H, with distinct amino acid changes observed at positions G448, G138, M220, and N266 [22]. In addition, the S240A mutation in *CYP51C*, which is associated with voriconazole resistance in *A. flavus*, has also been found to correspond to substitutions at the analogous position in the *CYP51* homologs of *Fusarium*, albeit with different amino acid changes [72]. However, the impact of these single nucleotide polymorphisms (SNPs) on azole resistance in *Fusarium* warrants further in-depth investigation, particularly with respect to *CYP51C* mutations located in non-conserved regions of the *CYP51* homologs in *Fusarium*. Furthermore, the normal function of certain key genes is critical for maintaining intrinsic resistance to azoles in *Fusarium*. For example, in *F. oxysporum*, deletion of the NADPH-cytochrome P450 reductase gene *CPR1* significantly reduces ergosterol levels and affects the expression of downstream *CYP51* genes, thereby increasing susceptibility to multiple azoles [73]. In *F. graminearum*, the transcription factor FgAtrR has been shown to influence tolerance to azoles by directly regulating the expression of *FgCYP51A* and multiple ABC transporter genes [74]. These findings establish CPR1 and FgAtrR as key components of intrinsic azole resistance in *Fusarium*.

The opportunistic pathogen *F. solani* exhibits intrinsic resistance to echinocandins, including caspofungin and micafungin, which inhibit cell wall synthesis. This resistance originates from structural differences in its Fks1 target site. Studies have demonstrated that a phenylalanine-to-tyrosine substitution at position 639 (F639Y) in hot spot 1 of the Fks1 protein in *F. solani* reduces susceptibility to echinocandins by 4- to 8-fold in *Saccharomyces cerevisiae*. This suggests that the intrinsic sequence of Fks1 is a key determinant of intrinsic resistance to echinocandins in *Fusarium* [75].

#### 4.1.3. Intrinsic Resistance of *Mucorales*

In *Mucorales* fungi, the lanosterol 14α-demethylase (CYP51/Erg11) is encoded by two paralogous genes, designated *F1* and *F5*. Of these, a naturally occurring tyrosine-to-phenylalanine substitution (Y129F) disrupts the water-mediated hydrogen-bonding network required for drug binding, thereby preventing short-tailed azoles from effectively inhibiting the enzyme and conferring intrinsic resistance [76,77]. Furthermore, studies on *M. lusitanicus* have revealed that three paralogous genes of *erg6* (encoding sterol 24-C-methyltransferase) enable *Mucorales* fungi to switch to alternative sterol biosynthesis pathways upon azole treatment, producing non-toxic alternative sterols that alleviate azole-mediated inhibition and thereby confer resistance [78]. Another example of gene duplication as a strategy for antifungal resistance in *Mucorales* is the high copy number of homologs encoding drug efflux pumps. In *M. lusitanicus*, eight ABC transporters have been identified, among which *pdr1* is overexpressed in the presence of azoles. Deletion of *pdr1* not only enhances susceptibility to azoles but also induces increased expression of two additional ABC transporter genes, *pdr2* and *pdr6*. The compensatory interplay among these distinct efflux pumps cooperatively alleviates azole-mediated inhibition, thereby conferring resistance [79].

*Mucorales* (using *M. lusitanicus* as an example) possess dual resistance mechanisms against echinocandins, such as micafungin. First, the presence of three copies of the *fks* gene (encoding β-(1,3)-D-glucan synthase) is a key determinant of resistance. In addition, the calcineurin pathway is also involved in regulating the expression of *fksA* and *fksB*, further enhancing the resistance phenotype [80].

#### 4.1.4. Intrinsic Resistance of *Scedosporium* spp./*L. prolificans*

*Scedosporium* spp. and *L. prolificans* are renowned for their extreme multidrug resistance, exhibiting intrinsic resistance to multiple classes of antifungal agents, including azoles, AmB, and echinocandins. Unlike *A. fumigatus*, which contains two paralogous *CYP51* genes, the genomes of *Scedosporium* spp. and *L. prolificans* encode only a single CYP51 protein, which phylogenetically clusters within the CYP51B group [81]. Alignment of the CYP51 amino acid sequences from *Scedosporium* spp. and *L. prolificans* with that of CYP51A from *A. fumigatus* has revealed that residues at three positions (S153, I235, and A302) correspond to residues (G138, M220, and T289, respectively) that have been previously implicated in azole resistance in *A. fumigatus* [82]. These naturally occurring residue substitutions reduce the binding affinity of azoles to the target enzyme, thereby constituting the molecular basis of intrinsic resistance. Furthermore, in *S. apiospermum*, two species-specific amino acid substitutions (Y136F and G464S) have been identified, which correspond to mutations (Y121F and G448S, respectively) that are associated with azole resistance in *A. fumigatus* [81]. However, in *Scedosporium* spp. and *L. prolificans*, the same tandem repeat sequences in the 5′ upstream region of the *CYP51* gene that have been identified in *A. fumigatus* have not been detected [81,82].

Similarly, alignment of the Fks1 (β-1,3-glucan synthase catalytic subunit) amino acid sequence from *L. prolificans* with that of *S. cerevisiae* has revealed two intrinsic amino acid residues (F639Y and W695F) that have been previously reported to confer echinocandin resistance. Furthermore, three additional amino acid changes (D440A, S634R, and H1245R) have been identified outside the hotspot regions; however, their contribution to echinocandin resistance warrants further investigation [82].

### 4.2. Acquired Resistance: The Genome’s Adaptive Counterattack

Acquired resistance poses the greatest challenge in clinical practice. The underlying mechanisms are diverse, ranging from point mutations to global transcriptional reprogramming. Among filamentous fungi, the mechanisms of acquired azole resistance has been most extensively characterized in *A. fumigatus*, far more so than in other species. Meanwhile, *Fusarium* spp., *Mucorales*, and *Scedosporium* spp./*L. prolificans* have each evolved distinct adaptive strategies of their own.

#### 4.2.1. *Aspergillus* spp.

##### Target-Gene-Dependent Resistance Mechanisms: Mutations and Overexpression of *CYP51*

**Figure 2:**
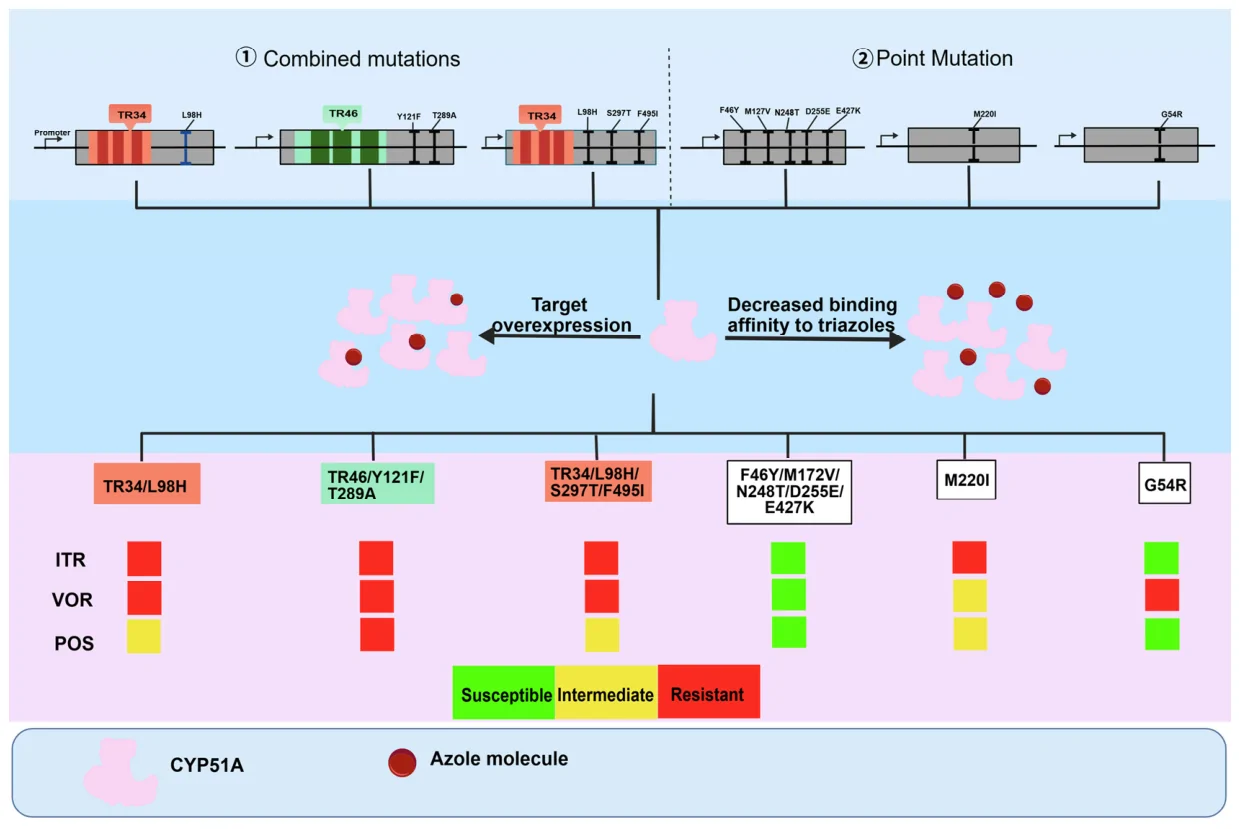
Impact of *CYP51A* mutations on azole susceptibility in *A. fumigatus*. The figure illustrates the spectrum of *CYP51A* mutations and their associated susceptibility profiles to triazole antifungals. The combinations of mutations, including TR34/L98H, TR46/Y121F/T289A, TR34/L98H/S297T/F495I, and the multi-site substitutions F46Y/M127V/N248T/D255E/E427K, as well as single mutations such as M220I and G54R, are shown. These mutations lead to either target overexpression or reduced binding affinity of the triazole molecules to the CYP51A enzyme. The susceptibility levels (susceptible, intermediate, or resistant) to itraconazole (ITR), voriconazole (VOR), and posaconazole (POS) are indicated by color-coded bars. The color gradient (green to red) reflects the progressive decrease in susceptibility, with red indicating resistance. This mutation-resistance correlation is critical for understanding clinical treatment failure and guiding antifungal therapy.

Target gene mutations and overexpression represent the most prominent and best-characterized mechanisms underlying acquired resistance. *A. fumigatus* possesses two CYP51 sterol 14α-demethylases, namely CYP51A and CYP51B. However, to date, only variations in the *CYP51A* gene and its protein product have been unequivocally linked to azole resistance (Figure 2) [83]. Mutations at specific loci in the *CYP51A* gene, such as G54, G138, M220, and G448, lead to key amino acid substitutions that alter the active-site pocket structure of the 14α-demethylase, thereby reducing the binding affinity of the drug to its target. This enables fungal cells to survive and proliferate in the presence of azoles. Such mutations have been widely reported in clinical isolates and are associated with cross-resistance to multiple azoles, directly impacting patient prognosis [84,85,86,87]. Tandem repeats (TR) in the promoter region of the *CYP51A* gene can significantly enhance gene transcription levels, leading to the overproduction of the target enzyme in cells. Typical TR-mediated resistance genotypes include TR34/L98H and TR46/Y121F/T289A, which are characterized by the insertion of 34bp or 46bp tandem repeats in the promoter region, respectively, accompanied by mutations in the coding region. These alterations result in cross-resistance to multiple azoles [88,89]. Transcription factors SrbA and AtrR have been shown to bind to the TR34 sequence, thereby inducing elevated expression of the *CYP51A* gene, which in turn may contribute to enhanced resistance to triazoles [89,90].

Among azole-resistant environmental isolates of *A. fumigatus*, TR34/L98H and TR46/Y121F/T289A are the predominant genotypes. The highest prevalence of TR34/L98H is found in India (88.9%), followed by Europe (77%), North America (58.8%), East Asia (37.4%), the Middle East (37.1%), Africa (29.3%), and South America (4.3%). In contrast, TR46/Y121F/T289A is frequently detected in South America, accounting for 60% of azole-resistant environmental isolates. It is also found in East Asia (25.3%), North America (23.5%), Europe (12.3%), Africa (9.8%), India (9.5%), and the Middle East (1.9%). Notably, South America is the only region where TR46/Y121F/T289A is more common than TR34/L98H. This may be explained by regional differences in crop types or the use of specific azole fungicides. By contrast, point mutations such as G54 are less frequent, with an estimated global prevalence of 1.8% in environmental samples. Nevertheless, their emergence during long-term azole therapy underscores the need for routine susceptibility testing in high-risk patients [23].

In addition, although studies remain relatively limited, *CYP51* gene mutations associated with azole resistance have also been progressively reported in other *Aspergillus* spp. Notably, an M217I mutation in the *CYP51Ap* gene has been identified in azole-resistant clinical isolates of *A. terreus* [91]. In addition, multiple novel mutations—S196F, A324P, N423D, V465M, and Y319H—have been identified in the *CYP51C* gene of voriconazole-resistant clinical isolates of *A. flavus* [92,93]. These findings suggest that target-gene mutations leading to acquired resistance are, to a certain extent, conserved within the genus *Aspergillus*, and that such mutations play a critical role in determining antifungal treatment outcomes.

##### Non-Target-Gene-Dependent Resistance Mechanisms

Multiple resistant strains that harbor completely wild-type *CYP51A* genes and their promoter regions nevertheless exhibit azole resistance, indicating the existence of non-target-gene-dependent resistance mechanisms in *A. fumigatus*. HapE, a subunit of the CCAAT-binding complex (CBC), has been identified as a key player in this context. The P88L mutation in HapE leads to the loss of the transcriptional repressive function of the CBC on *CYP51A*, thereby resulting in *CYP51A* overexpression and consequently conferring pan-azole resistance. This mechanism has been confirmed in clinical isolates, and its causal relationship with azole resistance has been validated through both homologous gene replacement and sexual crossing experiments [94]. HMG-CoA reductase hmg1 is the rate-limiting enzyme of the mevalonate pathway, which lies upstream of ergosterol biosynthesis. Studies have identified mutations in the *hmg1* gene—such as S305P, I412S, and a deletion of F262—in certain clinical resistant isolates, all of which are located within its sterol-sensing domain. Introduction of these mutations into susceptible strains results in elevated azole MICs without affecting *cyp51A* expression, suggesting that this mechanism operates independently of target enzyme overexpression. Sterol profiling has further revealed that the accumulation patterns of ergosterol precursors are altered in strains carrying *hmg1* mutations, potentially influencing drug susceptibility through modifications in membrane composition [95].

In addition to dysregulation of sterol biosynthesis resulting from transcriptional or enzymatic alterations, active efflux of azoles constitutes another common resistance mechanism. In certain clinical resistant isolates, consistent overexpression of ABC transporters—such as *cdr1B* (also designated as *abcB*/*abcC*/*abcG1*) and *atrF*—has been detected. These transporters actively pump azoles out of the cells, thereby reducing intracellular drug concentrations and conferring resistance [96,97]. However, the precise prevalence of efflux-driven resistance among clinical isolates remains undefined, and its relative contribution to clinical failure is less well established than that of CYP51A mutations, largely due to the lack of standardized detection methods and confirmatory clinical outcome studies. Furthermore, several putative multidrug resistance (MDR) transporters have also been implicated in triazole resistance, including Mdr1, Mdr2, Mdr3, and Mdr4. In resistant strains, some of these transporters exhibit constitutive overexpression, whereas others are induced upon exposure to triazoles. However, their specific contributions to resistance remain to be further validated through genetic approaches [96,98].

Genomic plasticity enables fungi to adapt to various stress factors and similarly contributes to their capacity to develop adaptive responses to antifungal agents. Deletion of the genes encoding the kinases AtmA and AtrA, which are involved in the DNA damage response, has been shown to enhance the adaptive evolutionary capacity of *A. fumigatus* under azole stress, leading to increased azole tolerance. These findings suggest that the functional integrity of these pathways plays an important role in maintaining genomic stability and restricting the emergence of resistance [99]. Deletion of the gene encoding the DNA mismatch repair protein MshA leads to genomic instability, conferring a higher evolutionary capacity upon exposure to posaconazole and ultimately resulting in the emergence of resistance. Moreover, variants of the *mshA* gene have also been detected in both clinical and environmental isolates [100]. In addition to the mechanisms described above, several other processes have also been proposed to be potentially associated with azole resistance, including biofilm formation, mitochondrial dysfunction, epigenetic alterations, RNA interference, metabolic network rewiring, and impaired drug penetration resulting from cell wall changes. However, the precise contributions of these factors and their mechanistic links to resistance require further investigation [101].

#### 4.2.2. *Fusarium* spp.

##### Mutations and Overexpression of the *CYP51* Gene

*Fusarium* spp. are intrinsically resistant to multiple azoles; however, under selective pressure from prolonged or prophylactic azole therapy, acquired mutations can further reduce their susceptibility. Target gene mutations or overexpression represent common resistance mechanisms to azoles in *Fusarium* spp. Laboratory-induced metconazole-resistant strains also harbor multiple amino acid substitutions, including G443S and D243N in CYP51A, or combined mutations such as E103Q and V157L. Moreover, in none of the mutant strains were changes observed in the promoter regions of the *FgCYP51* genes or in the *FgCYP51B* and *FgCYP51C* coding sequences, whereas the expression profiles of these genes were altered. For example, in mutants carrying the D243N substitution, all three *CYP51* genes exhibited overexpression; in the combined mutant (E103Q/V157L), only *CYP51A* and *CYP51B* were overexpressed. In contrast, in mutants harboring the G443S amino acid substitution, only *CYP51A* showed overexpression [102]. The Y137H substitution in CYP51B reduces the binding affinity of the target enzyme for tebuconazole, thereby compromising its inhibitory efficacy and conferring resistance to tebuconazole in *F. graminearum* [103]. In laboratory-induced hexaconazole-resistant mutants, a large number of amino acid substitutions were identified in the target genes, among which S28L, S256A, and V307A in CYP51C were consistently present in all resistant mutants [104]. A 23 bp deletion in the *CYP51A* promoter region was identified in nine voriconazole-resistant isolates of the *F. solani* species complex (FSSC); however, the underlying mechanism remains unclear, as this deletion resulted in only a 1.3-to 7.5-fold upregulation of *CYP51A* expression [105]. In addition, several studies have reported that transcription factors can influence azole resistance by regulating the expression of target genes. For example, azoles activate the Hog1-MAPK signaling pathway, leading to the phosphorylation of the transcription factor FgSR, which in turn upregulates the expression of the *FgCYP51A*/*B*/*C* genes. This compensatory upregulation counteracts the enzyme inhibition imposed by azoles, thereby achieving metabolic tolerance [106]. The transcription factor FgCreA influences ergosterol biosynthesis and azole susceptibility in *F. graminearum* by regulating the transcription of *FgCyp51A* and *FgErg6A* [107]. The transcription factor FgAtrR has been suggested to modulate the expression of *FgCYP51A* and ABC transporters, thereby contributing to azole resistance [74].

##### Overexpression of Drug Efflux Pumps

Overexpression of drug efflux pumps (primarily ABC transporters and MFS transporters) constitutes another important mechanism of acquired resistance in *Fusarium* spp. In *F. graminearum*, activation of FgQdr2, a plasma membrane-localized H^+^ antiporter, significantly reduces the susceptibility of the fungus to azole drugs [108]. FgABC3, FgABC4, and FgABCC9 have also been associated with resistance to azole drugs [109,110]. In *F. culmorum*, laboratory-induced adapted strains exhibited cross-resistance to azole drugs, and RNA-seq analysis revealed that the expression level of the pleiotropic drug resistance transporter FcABC1 (FCUL_06717) was up to 30-fold higher in response to tebuconazole treatment compared with the wild-type strain. This role of the transporter in triazole resistance was further corroborated by field isolates [111]. In *F. solani*, inhibition of efflux pump activity by promethazine led to increased susceptibility to voriconazole and AmB [112]. In addition, several studies have reported that transcription factors can influence azole resistance by regulating the expression of drug efflux pumps. For example, in *F. graminearum*, FgCrz1 translocates to the nucleus in a calcineurin-dependent manner upon tebuconazole treatment and regulates the expression of a range of non-*CYP51* genes, including multidrug transporters and membrane lipid-associated genes, thereby contributing to resistance. The high-osmolarity glycerol (HOG) signaling pathway cooperates with the calcium–calcineurin signaling pathway to modulate susceptibility to tebuconazole and pathogenicity. Notably, deletion of *FgCrz1* results in elevated phosphorylation levels of FgHog1 upon tebuconazole induction, which compensates for its regulatory role in resistance [113].

##### Biofilm: A Community-Level Fortress of Resistance

Biofilms are structured communities composed of orderly arranged microbial cells attached to living or non-living surfaces, surrounded by an extracellular matrix (ECM) consisting of polymeric substances. They exhibit substantially higher tolerance to antifungal agents than their planktonic counterparts [114]. *Fusarium* spp. have been demonstrated to form biofilms both in vitro and in vivo. The ECM of these biofilms physically impedes drug penetration, exposing cells in the deeper layers to subinhibitory concentrations and thereby leading to phenotypic resistance. This microenvironment also provides selective pressure that facilitates the evolution of higher-level resistance through mutations or adaptive gene expression. For example, biofilm formation in *F. solani* has been shown to elevate the MICs of natamycin, caspofungin, AmB, and voriconazole [115]. Proteomic analysis revealed that 19 proteins are overexpressed in biofilms, including pentitol enzymes, enolase, phosphoglycerate kinase, and ATP-citrate synthase, suggesting that glycolysis and gluconeogenesis play important roles during biofilm formation [116]. These changes may lead to metabolic and structural alterations in the cells, which in turn may contribute to the development of drug resistance. Moreover, fungi can secrete quorum sensing molecules (QSMs) to communicate between cells and coordinate the overall behavior of the community, a process that is crucial for biofilm formation [117]. In summary, *Fusarium* spp. may act in concert through the physical barrier of the ECM, metabolic heterogeneity of internal cells, and quorum sensing, collectively leading to a marked increase in drug resistance.

#### 4.2.3. *Mucorales*

*Mucorales* develop acquired resistance to the antifungal agent FK506 (a calcineurin inhibitor) through two distinct mechanisms. One mechanism involves classical genetic mutations that confer stable drug resistance, whereas the other operates through an epigenetic pathway leading to unstable drug resistance. In the genetic mutation pathway, mutations in key genes, including the drug receptor and target, have been identified as contributing to resistance. For example, in resistant isolates selected under FK506 pressure, mutations have been identified in the *fkbA* gene (encoding FKBP12), the calcineurin catalytic subunit genes (*cnaA*/*cnaB*), and the regulatory subunit B gene (*cnbR*), resulting in a hyphal growth phenotype rather than yeast-like growth [118,119]. Furthermore, mutations in *bycA* (encoding an amino acid permease) enable the strain to bypass calcineurin and restore hyphal growth, conferring resistance to both FK506 and cyclosporin A (CsA) [120].

Furthermore, the reversible, non-genetic resistance mediated by epigenetic pathways represents a distinctive acquired resistance mechanism in *Mucorales*. In *M. bainieri*, the majority (90%) of FK506-resistant isolates exhibit unstable and transient resistance, readily reverting to drug susceptibility upon passage in the absence of the drug, without associated DNA mutations. In half (50%) of the isolates, FK506 resistance is conferred by RNA interference-dependent epimutations, in which small interfering RNAs (siRNAs) post-transcriptionally silence *fkbA* gene mRNA, leading to reduced mRNA and protein levels of FkbA [121]. This mechanism has been validated in in vivo infection models and can occur in different organs [122]. The same RNAi-mediated epimutation pathway is also involved in the development of resistance to 5-fluoroorotic acid (5-FOA), in which elevated levels of small RNAs (sRNAs) targeting the *pyrF* gene (encoding orotate phosphoribosyltransferase) and the *pyrG* gene (encoding orotidine-5′-phosphate decarboxylase) reduce the levels of these two enzymes, thereby preventing the conversion of 5-FOA into its toxic derivative and conferring resistance [123]. The remaining majority (40%) of FK506-resistant isolates achieve resistance through transcriptional repression of *fkbA* and its neighboring genes via heterochromatin silencing mediated by H3K9 dimethylation [121]. The non-canonical RNAi pathway (NCRIP) negatively regulates the epimutation pathway. Knockout of key NCRIP genes, such as *r3b2* and *rdrp1*, significantly increases the frequency of epimutant emergence, leading to resistance [124].

#### 4.2.4. *Scedosporium* spp./*L. prolificans*

Studies on the resistance mechanisms of *Scedosporium* spp. and *L. prolificans* have been largely focused on intrinsic resistance conferred by differences in drug targets, whereas acquired resistance mechanisms have been less extensively studied and are primarily confined to adaptive responses following azole treatment. Upon exposure to voriconazole, *L. prolificans* exhibits marked morphological changes, with hyphae becoming significantly shorter and wider. While total cell area remains largely unchanged, cell wall thickness increases notably, particularly the outer fibrous layer, which thickens by nearly 3-fold. Further analysis revealed that the carbohydrate composition of cell wall is altered in the presence of voriconazole, with marked increases in glucose and mannose content and induced enhancement of chitin synthesis. In addition, adaptive responses at the proteomic level have been observed, including upregulation of Srp1 family-like protein (Serine-rich RNA polymerase I suppressor protein) and heat shock protein 70 (Hsp70) [125]. These findings suggest that the voriconazole-induced morphological alterations, cell wall remodeling, and proteomic adaptive responses may be directly or indirectly associated with antifungal resistance in *L. prolificans*; however, the specific functional contributions of these changes warrant further investigation.

*Scedosporium* spp. and *L. prolificans* form typical biofilms that render treatment particularly challenging, and the biofilm-associated resistance results from the synergistic action of multiple mechanisms. The ECM acts as a physical and chemical barrier that binds and sequesters drugs, impeding their penetration to fungal cells within the deeper layers of the biofilm and thereby reducing the effective drug concentration. During biofilm formation, the activity of drug efflux pumps in fungal cells is significantly higher than that in planktonic spores, actively extruding drugs out of the cells and decreasing intracellular drug concentrations. Biofilm cells exhibit intrinsically higher basal activity of antioxidant enzymes, including SOD and CAT, which effectively scavenge ROS induced by antifungal agents such as amphotericin B, thereby counteracting oxidative killing. In addition, the mitochondrial AOX plays a critical protective role in biofilms by modulating ROS levels and helping cells tolerate drug-induced stress [126]. In-depth investigation of these mechanisms provides a clear direction for the development of novel therapeutic strategies, such as combination therapy targeting the ECM, efflux pumps, AOX, or the antioxidant system.

For *Fusarium* spp., *Mucorales* and *Scedosporium* spp./*L. prolificans*, intrinsic resistance is universal, and acquired target-site mutations are rarely reported in clinical isolates. Consequently, clinical management of these infections depends more on antifungal susceptibility testing-guided combination therapy than on genotypic resistance markers, which remain largely investigational [127].

## 5. Advances in Molecular Diagnostics and Resistance Detection

Early and accurate diagnosis of filamentous fungal infections, together with rapid identification of antifungal resistance, is critical for implementing effective antifungal therapy and improving patient outcomes. Although conventional culture-based methods and phenotypic antifungal susceptibility testing remain the gold standard, they are time-consuming (requiring several days to weeks), exhibit limited sensitivity, and have clear limitations for slow-growing or difficult-to-culture fungi. With the rapid advances in molecular biology and genomics, diagnostic approaches are shifting from reliance on in vitro culture toward direct analysis of pathogen genetic material and rapid phenotypic assessment. This chapter summarizes the currently available molecular and non-molecular detection tools, evaluates their clinical utility, advantages, and limitations, and outlines their clinical application strategies and future directions (Table 1).

### 5.1. Traditional Phenotypic Detection

The broth microdilution method directly reflects the resistance phenotype by measuring fungal growth inhibition in the presence of antifungal agents, and its results exhibit a straightforward correlation with clinical outcomes. The European Committee on Antimicrobial Susceptibility Testing (EUCAST) and the Clinical and Laboratory Standards Institute (CLSI) have published standardized broth microdilution guidelines for filamentous fungi (EUCAST-E.Def 9.4 and CLSI-M38). These documents provide detailed specifications regarding medium (RPMI 1640), inoculum concentration, incubation conditions, and endpoint reading, with the aim of improving inter-laboratory reproducibility and providing baseline data for epidemiological surveillance [128]. In terms of result interpretation, epidemiological cut-off values (ECV/ECOFF) are used to distinguish “wild-type” strains from “non-wild-type” strains that may harbor resistance mechanisms, and serve as the primary tool for surveillance of emerging resistance. The ECOFF for a wide range of moulds are explicitly listed in official EUCAST documents. In contrast, clinical breakpoints (CBP) integrate pharmacokinetic data and treatment outcomes to guide therapeutic decision-making. However, for filamentous fungi, species-independent breakpoints cannot be established for *Aspergillus* due to insufficient clinical data and biological complexity. Currently, species-specific breakpoints have been defined for certain species, such as *A. fumigatus* with voriconazole, for which ≤1 mg/L is considered susceptible and >1 mg/L resistant. The E-test is convenient and shows good correlation with broth microdilution MICs and is often used in clinical laboratories as an alternative or supplementary method. However, owing to difficulties in standardization, it is not recommended as the first-line choice for routine antifungal susceptibility testing [129].

### 5.2. Nucleic Acid-Based Molecular Diagnostics

Nucleic acid-based molecular diagnostics can provide species identification and clues to resistance either without the need for culture or at an early stage of culture, significantly shortening turnaround time and improving the detection rate of fastidious pathogens. However, their results must be interpreted in conjunction with phenotypic and clinical data. ITS region sequencing remains the international standard for fungal species identification and is particularly useful for morphologically indistinguishable or atypical isolates. For *Aspergillus* spp., β-tubulin (*benA*) or calmodulin gene sequencing offers higher resolution for species-level identification [130]. Real-time fluorescent PCR involves primers and probes designed to target conserved genes of specific pathogens, such as the clinically important *Aspergillus* spp. (*A. fumigatus*, *A. flavus*, *A. niger*, and *A. terreus*) and enables highly sensitive, specific, and rapid detection directly from clinical specimens [131]. A multiplex real-time quantitative PCR (M-qPCR) assay based on TaqMan probes has been successfully developed for the simultaneous detection of four clinically important filamentous fungi: *A. fumigatus*, *Fusarium* spp., *Mucorales*, and *Histoplasma capsulatum*, with 100% specificity and high sensitivity [132].

Targeted detection represents the most successfully translated molecular resistance assay in clinical practice, particularly for azole resistance in *A. fumigatus*. By designing specific primers or probes targeting known hotspot mutations in *CYP51A* (such as TR34, L98H, Y121F, T289A, G54, and M220), these key resistance mutations can be detected directly from clinical specimens or cultured isolates within hours, providing rapid evidence for clinical decision-making [133,134]. Commercial detection kits developed using this technology have been adopted in some laboratories [135]. In addition, when combined with sequencing, this approach enables the detection of both known and unknown point mutations as well as promoter insertions, offering greater comprehensiveness than targeted PCR, albeit with a slightly longer turnaround time and higher demands on data analysis [133].

Metagenomic next-generation sequencing (mNGS) enables high-throughput sequencing of all nucleic acids extracted from clinical specimens, allowing unbiased detection of all microorganisms present without the need for culture. This approach offers substantial value in cases where conventional methods yield negative results, in mixed infections, or when rare or unusual pathogens are suspected. However, it also has limitations, including high background noise, complex data analysis, high cost, and limited depth of coverage for resistance genes [136]. Whole-genome sequencing (WGS) has been widely employed to analyze the genetic diversity of filamentous fungi, such as *A. fumigatus*, and its association with antifungal resistance, and is regarded as the ultimate tool for investigating resistance mechanisms and molecular epidemiology. It comprehensively reveals all potential resistance-associated mutations (e.g., in *CYP51A* and beyond), and enables precise tracking of healthcare-associated or regional outbreaks and clonal transmission through SNP analysis. WGS is progressively transitioning from a research tool to clinical application, particularly for resolving complex resistance issues and outbreak investigations [137,138].

### 5.3. Emerging Detection Technologies

Matrix-assisted laser desorption/ionization time-of-flight mass spectrometry (MALDI-TOF MS), with its high-throughput, high-accuracy, and rapid (within minutes) identification capability, has become a routine tool for bacterial and yeast identification in clinical microbiology laboratories. Through the expansion of reference databases, its capacity for the identification of filamentous fungi has been enhanced, and it can serve as a complementary approach to conventional fungal identification, thereby improving the accuracy of species-level identification [139,140]. Preliminary progress has also been made in the application of MALDI-TOF MS to resistance detection. Studies have shown that, when combined with protein peak pattern analysis, this technique is capable of distinguishing species within the *A. fumigatus* complex, detecting azole-resistant *A. fumigatus* isolates, and even discriminating between strains carrying different *CYP51A* mutations. However, these studies remain at an early stage, and a broadly applicable clinical protocol has not yet been established [141].

Droplet microfluidic platforms are capable of encapsulating individual fungal cells in microdroplets and exposing them to varying concentrations of antifungal agents, enabling rapid assessment of growth inhibition through microscopic detection (e.g., within 24 h), thereby substantially shortening the turnaround time for phenotypic antifungal susceptibility testing. In one study, single spores of the filamentous fungus *Alternaria alternata* were encapsulated in droplets, and their germination and hyphal growth were monitored in real time to evaluate resistance. The results showed a high correlation with conventional methods, demonstrating the feasibility of this approach as a rapid susceptibility testing tool. This platform allows the analysis of over 2000 droplets in a single experiment, and when combined with automated image analysis, can further reduce the analytical cycle time. In addition, the platform can be extended to other filamentous fungi by adjusting droplet size, culture medium, and incubation conditions, and also supports combination drug screening, offering a highly promising technological solution for rapid antifungal resistance research in filamentous fungi [142].

In the field of serological and antigen detection, galactomannan (GM) and β-D-glucan (BDG) assays play important adjunctive roles in the early diagnosis and therapeutic monitoring of invasive fungal diseases. The GM test, together with PCR, is primarily used for the early diagnosis and monitoring of treatment response in invasive aspergillosis. Although these assays do not directly detect resistance, dynamic monitoring of GM index or fungal burden (via quantitative PCR) can be used to indirectly assess treatment response. Persistent elevation of GM levels despite apparent therapeutic intervention may indicate the presence of a resistant infection and warrants further investigation [143]. BDG is a common component of the cell wall in a wide range of fungi (with the exception of *Cryptococcus* and *Mucorales*) and can serve as a pan fungal biomarker for the early screening and therapeutic monitoring of invasive aspergillosis (IA), invasive candidiasis (IC), and other invasive fungal infections (IFI), excluding mucormycosis and cryptococcosis [144].

### 5.4. Clinical Application Strategies and Future Challenges

An ideal clinical pathway should employ a tiered, synergistic combination of multimodal diagnostic methods. For critically ill patients, rapid non-culture screening tests (e.g., GM/BDG and real-time PCR) should be prioritized for early diagnosis. Once isolates are obtained, MALDI-TOF MS enables rapid species identification, followed by standardized phenotypic susceptibility testing. For cases with suspected *A. fumigatus* infection, targeted *CYP51A* molecular testing can be performed concurrently to provide early resistance alerts. For complex cases or suspected outbreaks, WGS is recommended to elucidate resistance mechanisms and conduct epidemiological tracing.

The main challenges are summarized as follows. First, molecular testing—particularly mNGS and quantitative PCR—urgently requires standardization in sample processing, nucleic acid extraction, testing protocols, and result interpretation. Second, direct detection of resistance mutations from clinical specimens remains less sensitive than from cultured isolates, calling for improved nucleic acid enrichment and amplification strategies. Third, molecular resistance markers for non-*A. fumigatus* pathogens, such as *Fusarium* spp. and *Mucorales*, remain poorly characterized. Fourth, the development of point-of-care testing (POCT) devices remains a long-term objective, particularly for resource-limited settings and ICU bedside applications.

## 6. Clinical Therapeutic Strategies and Challenges

The emergence and spread of antifungal resistance in filamentous fungi have fundamentally undermined the cornerstone of treatment for invasive fungal infections. When standard therapeutic regimens fail as a result of resistance, patients face a marked increase in mortality rates. Therefore, formulating and optimizing clinical therapeutic strategies based on an in-depth understanding of resistance mechanisms lies at the heart of addressing this crisis.

### 6.1. Optimizing the Use of Existing Antifungal Agents

Therapeutic drug monitoring (TDM), which involves measuring drug concentrations in patients’ blood to individualize dosing, is a critical tool for achieving effective therapy while minimizing toxicity, particularly for drugs with substantial interindividual pharmacokinetic variability. The pharmacokinetics of voriconazole are influenced by multiple factors, including *CYP2C19* polymorphisms, critical illness, host immune status, and site of infection. A large body of evidence indicates that maintaining voriconazole trough concentrations within the range of 1–5.5 mg/L provides an optimal balance between clinical efficacy and toxicity [145]. Isavuconazole, owing to its more predictable pharmacokinetic profile, has a lower requirement for routine TDM; however, it remains valuable in specific situations, such as in patients with extreme body weight, gastrointestinal absorption disorders, or severe hepatic/renal impairment [146]. For echinocandins, the evidence base for routine TDM is relatively weak; however, monitoring plasma concentrations may be beneficial for optimizing efficacy in specific patient populations, such as those with obesity, critical illness, or treatment failure [147].

When monotherapy is inadequate, combination therapy with agents of different mechanisms of action represents an important strategy for overcoming resistance. The rationale is based on synergistic effects, which may overcome specific resistance mechanisms and reduce the selection of resistant mutants. Clinicians commonly combine azoles with echinocandins as a standard regimen, particularly in cases of refractory aspergillosis. The underlying mechanism may involve echinocandin-induced disruption of the cell wall, thereby enhancing the penetration and uptake of azoles (which act on the membrane) [148]. Combination therapy with AmB and echinocandins has demonstrated synergistic effects and improved survival rates in the treatment of mucormycosis, as evidenced in both animal models and clinical reports [149,150]. According to the European Society of Clinical Microbiology and Infectious Diseases (ECMM) guidelines, combination therapy with voriconazole and terbinafine is strongly recommended as a first-line treatment for *Scedosporium* spp. infections, as previous studies have demonstrated higher survival rates and superior treatment outcomes with this regimen [151].

Adjunctive therapies, used in combination with antifungal agents, may improve outcomes in selected patients, and include immune reconstitution, surgical intervention, and novel adjunctive modalities. In neutropenic patients, rapid restoration of granulocyte counts is a decisive factor for treatment success. The use of granulocyte colony-stimulating factor (G-CSF) or granulocyte-macrophage colony-stimulating factor (GM-CSF) represents a viable therapeutic strategy [152]. For localized infections such as pulmonary aspergilloma, rhino-orbital-cerebral mucormycosis, and cutaneous fusariosis, aggressive surgical debridement or resection, combined with systemic antifungal therapy, can significantly improve cure rates and reduce the risks of recurrence and mortality [153]. The use of immunomodulatory agents, such as interferon-gamma and granulocyte colony-stimulating factor, has shown potential as adjunctive therapy in some studies, particularly in patients with primary immunodeficiencies such as chronic granulomatous disease who present with refractory infections [154].

### 6.2. Exploration of Novel Antifungal Agents and Targets of Action

#### 6.2.1. Next-Generation Azole and Echinocandin Antifungal Agents

Isavuconazole is a broad-spectrum triazole antifungal agent with efficacy comparable to that of voriconazole or amphotericin B, but with fewer drug-related adverse events. It has been approved by the U.S. Food and Drug Administration (FDA) and the European Medicines Agency (EMA) for the treatment of invasive aspergillosis and invasive mucormycosis when amphotericin B is not suitable. Its activity against *Mucorales* makes it an important therapeutic option for mucormycosis and broadens the antifungal spectrum of the azoles [155]. In addition, isavuconazole may retain some activity against certain azole-resistant *A. fumigatus* isolates, such as those harboring only point mutations like G54 or M220; however, it is generally ineffective against high-level resistance mutants, including the TR34/L98H genotype [156]. Oteseconazole is a novel triazole antifungal agent that was approved by the U.S. FDA in April 2022 for the treatment of recurrent vulvovaginal candidiasis (RVVC). Its therapeutic potential for other fungal infections, such as invasive candidiasis and aspergillosis, is currently under investigation. Opelconazole is an investigational inhaled azole antifungal agent in development, intended for the treatment of pulmonary fungal infections, particularly pulmonary aspergillosis [157].

Rezafungin is a novel echinocandin agent that exhibits excellent stability and solubility, as well as a uniquely long half-life that enables once-weekly dosing. Clinical data have shown that rezafungin has activity comparable to that of other echinocandins against *Candida* spp. and *Aspergillus* spp., including efficacy against echinocandin-resistant *Candida* spp. and azole-resistant *Aspergillus* spp. isolates [158]. In addition, rezafungin has also been shown to exhibit favorable in vitro activity against *A. terreus* and against cryptic species that are non-wild-type to AmB [159]. Available data have suggested that rezafungin has a favorable safety and efficacy profile, positioning it as a promising new candidate agent in the antifungal armamentarium.

#### 6.2.2. Novel Agents Targeting New Targets

Ibrexafungerp is a triterpenoid antifungal agent that inhibits glucan synthase at a different step from echinocandins and is available in an oral formulation. Available data indicate that it is active against echinocandin-resistant *Candida* species and also exhibits activity against *Aspergillus* spp., offering a new option for combination therapy. Fosmanogepix is the prodrug of manogepix and acts as an inhibitor of Gwt1, an enzyme in the glycosylphosphatidylinositol (GPI) anchor protein biosynthesis pathway. It has high oral bioavailability and demonstrates activity against a broad range of drug-resistant filamentous fungi, including *Aspergillus* spp., *Fusarium* spp., *Scedosporium* spp., and *R. arrhizus*, as well as against yeasts such as *Cryptococcus* and *Candida* spp. Olorofim is a novel pyrimidine synthesis inhibitor that shows activity against a range of hyaline moulds, including *Fusarium* spp., and holds promise for future clinical application [157].

#### 6.2.3. Non-Conventional Antifungal Strategies

Monoclonal antibodies, particularly those targeting fungal surface antigens such as β-glucan, can serve as adjuvants to antifungal agents, helping to prevent or clear fungal infections by enhancing both nonspecific and specific immune responses [160]. Biomimetic antifungal materials, including antifungal peptides, chitosan derivatives, and nanoparticles, function through mechanisms such as disruption of the cell membrane, generation of ROS, and inhibition of essential fungal processes [161]. Targeting virulence factors, such as the addition of iron chelators in combination with liposomal AmB, has demonstrated synergistic effects and improved survival rates in murine models of mucormycosis [162].

### 6.3. Management and Prevention Strategies for Antifungal Resistance

#### 6.3.1. Healthcare-Associated Infection Control and Antifungal Stewardship

Hospitals should strengthen environmental monitoring and control. In centers where nosocomial transmission of azole-resistant *A. fumigatus* has been documented, rigorous environmental investigations should be conducted, and effective air filtration and disinfection measures should be implemented. Isolation of patients colonized or infected with resistant strains, particularly those who are neutropenic, may help interrupt transmission. In the use of antifungal agents, unnecessary empirical therapy and prophylaxis should be restricted; antifungal agents should be prescribed rationally based on etiological findings and susceptibility profiles; and TDM-guided precision dosing should be promoted. The use of agents with confirmed high-level community resistance for prophylactic purposes should be avoided. These measures help reduce drug selection pressure and delay the emergence of hospital-acquired resistance [163].

#### 6.3.2. Regulation at the Agricultural and Environmental Level

The use of agricultural triazole fungicides is an important driver of environmental azole-resistant *A. fumigatus*. Accordingly, multi-sectoral coordination in regulatory oversight is necessary, and the specific measures are outlined below: (1) restricting the use of agricultural azoles that are structurally similar to clinical agents and for which cross-resistance has been demonstrated; (2) promoting integrated management strategies that reduce reliance on single chemical classes; (3) develop and apply non-azole agricultural fungicides with different modes of action; and (4) establishing cross-sector surveillance systems that link agricultural fungicide usage data with clinical resistance surveillance. Collaborative efforts between agricultural authorities, environmental agencies, and healthcare systems are essential to mitigate the environmental selection pressure that drives the global spread of azole-resistant *A. fumigatus* [34].

#### 6.3.3. Global and Regional Surveillance Networks

Establishing active surveillance systems is essential. The resistance rates, resistance mechanisms, and clonal transmission dynamics of filamentous fungi in clinical and environmental samples should be continuously monitored, and international resistance databases and rapid early warning platforms should be developed. Standardized antifungal susceptibility testing and molecular detection methods are fundamental to ensuring data comparability. When novel resistant clones or mechanisms are identified, alerts should be rapidly disseminated worldwide to guide clinical practice and public health responses [65].

## 7. Conclusions and Perspectives

**Figure 3:**
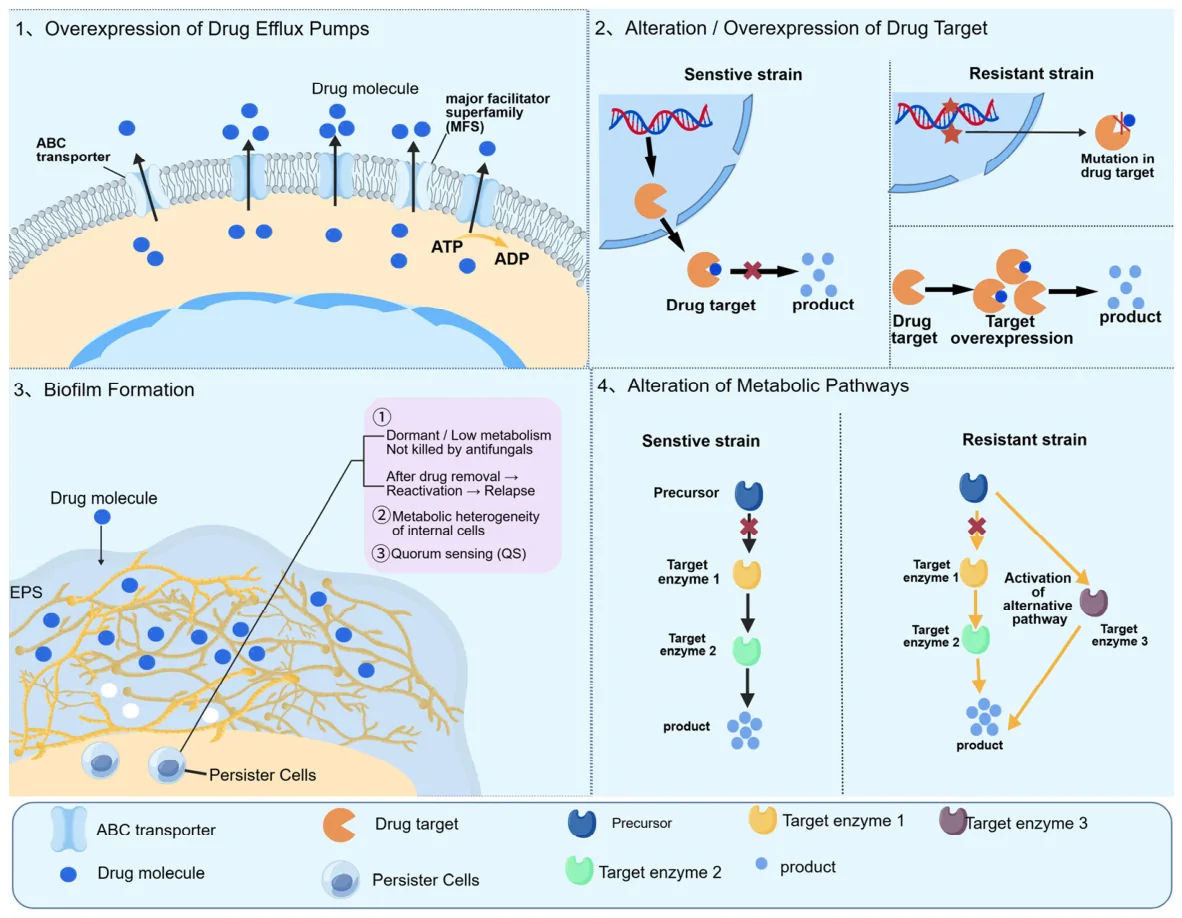
Overview of major antifungal resistance mechanisms in filamentous fungi. Schematic overview of the four primary strategies through which filamentous fungi evade the effects of antifungal drugs. (1) Overexpression of drug efflux pumps: Membrane-associated transporters, primarily belonging to the ATP-binding cassette (ABC) and major facilitator superfamily (MFS), actively extrude antifungal agents from the intracellular space, reducing intracellular drug concentrations to sublethal levels. (2) Alteration or overexpression of drug targets: Mutations in target genes (e.g., *CYP51A* in *A. fumigatus*, *FKS* hotspots in echinocandin-resistant isolates) reduce drug-binding affinity, while promoter tandem repeats (e.g., TR34/L98H, TR46/Y121F/T289A) drive target enzyme overexpression, collectively diminishing drug efficacy. (3) Biofilm formation: Fungal cells embedded in an extracellular polymeric matrix exhibit increased tolerance to antifungals through mechanisms such as physical diffusion barriers, reduced metabolic activity of persister cells, and quorum sensing molecules (QSMs). (4) Alteration of metabolic pathways: Adaptive rewiring of sterol biosynthesis (e.g., alternative ergosterol pathways in *Mucorales*), upregulation of stress response pathways (e.g., Hsp90, calcineurin, and Hog1-MAPK signaling), and metabolic compensation mechanisms enable fungal cells to bypass drug-induced inhibition. These mechanisms often act synergistically, leading to multidrug resistance and clinical treatment failure. Abbreviations: ABC, ATP-binding cassette; MFS, major facilitator superfamily; Hog, high-osmolarity glycerol; MAPK, mitogen-activated protein kinase.

Antifungal resistance in filamentous fungi has become a major global health threat. The underlying mechanisms are remarkably complex, involving both intrinsic and acquired resistance. Classical pathways—such as target gene mutations and efflux pump overexpression—coexist with emerging mechanisms, including epigenetic regulation and biofilm formation (Figure 3). Different pathogens exhibit distinct resistance profiles: azole resistance in *A. fumigatus* is the most pressing global concern, while *Fusarium* spp., *Mucorales*, and *Scedosporium* spp./*L. prolificans* are inherently multidrug-resistant and thus leave few therapeutic options.

Diagnostic approaches are shifting from conventional culture-based methods toward an integrated application of molecular and rapid phenotypic techniques. MALDI-TOF MS, targeted resistance gene PCR, and mNGS have markedly improved diagnostic efficiency, although challenges in standardization and sensitivity persist. Therapeutic strategies should be stratified according to susceptibility profiles and species identification. Precision dosing through TDM, combination regimens, and novel agents (such as isavuconazole, fosmanogepix, and olorofim) are progressively enhancing clinical outcomes.

Looking ahead, it is essential to deepen our understanding of resistance evolution and host microenvironment interactions in basic research; accelerate the development of diagnostic innovations at the technological level—including microfluidic susceptibility testing platforms that enable high-throughput and same-day phenotypic profiling, artificial intelligence-based predictive models that integrate genomic and clinical data for resistance prediction, and point-of-care testing devices for rapid detection directly from clinical specimens; promote the clinical validation of novel target-based agents and immunotherapies (e.g., checkpoint inhibitors, interferon-gamma, and monoclonal antibodies targeting fungal antigens) in translational research—that have shown encouraging results in preclinical models and early clinical case series of invasive mold infections, and curb the dissemination of resistant clones at the source through global surveillance and multisectoral collaboration.

## Figures and Tables

**Table 1:** Comparison of diagnostic methods for antifungal resistance detection in filamentous fungi.

Method	Turnaround Time	Sensitivity/Specificity	Resistance Detection	Cost	Key Limitations
Broth microdilution	*Mucorales* 24 h; most moulds 48 h; *Scedosporium* spp. 72 h	High/High (gold standard)	Direct (phenotypic)	Moderate (consumables cheap; labor-intensive)	Time-consuming; requires skilled personnel; affected by inoculum size, microplate material, and incubation conditions; MEC reading for echinocandins requires microscopy–labor-intensive
E-test	48 h	Moderate/Moderate-to-low (Species–drug dependent)	Direct (phenotypic)	Moderate (E-test strips)	Limited standardization; not first-line; poor agreement for some species
ITS Sequencing	Sanger 24–48 h; Illumina amplicon 2–3 days	Genus/section level: High; Species level: Low	Indirect (genotypic)	Moderate to high (costly per strain; batch-reduced)	Limited resolution; read-length limitation; database and annotation quality issues; primer/copy-number bias; no phenotypic correlation
M-qPCR	Hours (PCR + analysis)	High/High (for known mutations)	Indirect (genotypic)	Moderate (commercial kits higher)	Limited species and sample type coverage; novel mutations not detectable; requires skilled personnel
Targeted PCR (*CYP51A*)	Hours (PCR + analysis)	High/High (for known mutations)	Indirect (genotypic)	Moderate (commercial kits higher)	Detects only known mutations; no phenotypic correlation
mNGS	24–72 h	High (High variability across platforms)/High (specificity reduced in non-sterile specimens)	Indirect (genotypic)	Very high	High cost; complex data analysis; limited resistance gene coverage; requires combined interpretation with imaging; lacks standardized clinical analysis workflow
WGS	Days–weeks	High/High	Indirect (genotypic)	Very high	Complex data analysis; not routine; requires pure culture
MALDI-TOF MS	Minutes	High/High (depending on species, drug, and method)	Indirect (species/peak patterns)	High equipment cost; low per-sample cost	Limited resistance libraries; requires pure culture; research-stage for resistance
Droplet microfluidics	With 24 h	High/High (research-stage)	Direct (phenotypic)	High upfront investment; low per-assay cost	Emerging technology; limited validation in clinical settings; limited scope; microscopy-dependent with image analysis
GM/BDG assays	Hours	Moderate to high (High heterogeneity)	Indirect (biomarker)	Moderate (commercial kits higher)	No resistance detection; supportive only; high heterogeneity; limited to specific patient populations

## Data Availability

No new data were created or analyzed in this study. Data sharing is not applicable to this article.
